# Ancillary body movements in piano performance: a critical narrative review of structural coupling, audience cues, and cognitive-scaffolding claims

**DOI:** 10.3389/fpsyg.2026.1838025

**Published:** 2026-04-28

**Authors:** Yue Sun, Yilin Wang

**Affiliations:** 1 Saint Petersburg State Institute of Culture, Saint Petersburg, Russia; 2 Department of Culture, Communication & Media, Institute of Education, University College London, London, United Kingdom

**Keywords:** ancillary movements, audiovisual integration, cognitive scaffolding, embodied cognition, piano performance, visual dominance

## Abstract

Although piano performance is often treated as centered on sound production, it also involves body movements commonly discussed as ancillary, whose observable role extends beyond immediate sound production. These movements have often been discussed through broad embodied-cognition frameworks, yet the empirical basis for specific mechanistic claims remains uneven. This critical narrative review synthesizes research on movements commonly discussed as ancillary from two related but unevenly developed bodies of evidence: their reported relationships with musical organization in performers, and their role as visual cues in audience evaluation. On the performer side, kinematic findings suggest that movements commonly discussed as ancillary are often systematically related to phrasing, meter, and expressive organization, but direct evidence for cognitive-load reduction remains limited. On the audience side, audiovisual studies indicate that visible kinematics can substantially shape judgments of expressivity, emotion, and performance quality, although the strength of these effects varies across tasks and study designs. Across both domains, the literature remains constrained by small samples, limited ecological breadth, and an overreliance on correlational evidence. We therefore argue that current findings support the relevance of movements commonly discussed as ancillary as structural and perceptual cues, but do not yet justify strong claims that they function as cognitive scaffolds. Future work should combine controlled kinematic manipulation with multimodal measurement to clarify how these movements may simultaneously serve overlapping communicative, regulatory, and biomechanical functions.

## 1 Introduction

Although piano performance is often treated as being centered on sound production, it is inherently multisensory, involving tightly coupled auditory, visual, and motor processes (Davidson, 1993; Vines et al., 2006; Bishop and Goebl, 2014). In addition to key-striking actions, performance also entails a range of body movements—including torso sway, head motion, breathing-related adjustments, and facial expressions—that extend beyond immediate sound production (Davidson, 1993, 2007; Thompson and Luck, 2012; Sakaguchi et al., 2016). Following prior literature (e.g., Cadoz and Wanderley, 2000), this review adopts the term ancillary movements (AMs). However, we emphasize that this is used as an analytical and functional distinction rather than a strictly biomechanical one. Specifically, AMs are defined here as movements that are not primarily intended to produce sound, even though—due to intersegmental coupling and multijoint dynamics—they may still indirectly influence sound production. From a biomechanical perspective, it is difficult to isolate movements that are entirely unrelated to sound generation. For example, torso orientation can affect upper-limb kinematics and key-contact dynamics (Verdugo et al., 2020). Accordingly, the present review does not treat AMs as mechanically independent, but rather as movements whose observable role extends beyond immediate acoustic generation. While early pedagogy frequently dismissed these movements as mere showmanship or idiosyncratic habits, classic empirical studies have demonstrated that visual kinematics effectively transmit performance intentions, occasionally proving more informative than auditory cues for specific esthetic judgments (Davidson, 1993). Subsequent physiological and kinematic research has revealed that expressive playing systematically restructures upper limb and torso organization—an effect that transcends the mere byproduct of physical exertion (Nakahara et al., 2010; Turner et al., 2025). Concurrently, psychophysical studies indicate that audience evaluations of emotion and expressivity are often influenced by audiovisual cues (Vines et al., 2011). This review therefore retains the term primarily as a heuristic label for organizing the literature, rather than as a claim about a sharply separable biomechanical class of movements.

Rather than treating embodied cognition as a sufficient explanation, we adopt a more constrained usage of the term. Here, embodied cognition refers broadly to frameworks proposing that cognitive processes are grounded in bodily states and sensorimotor interactions (e.g., Wilson, 2002; Barsalou, 2008). Importantly, we distinguish between broad theoretical frameworks of embodiment and specific mechanistic claims, the latter requiring clearly specified and empirically testable causal pathways. This article offers a critical narrative review of the literature on ancillary movements in piano performance. It reviews two related but unevenly developed bodies of evidence. First, to what extent does existing performer-side evidence support claims that movements often classified as ancillary contribute to phrasing, timing, or cognitive offloading? Second, how do audience studies characterize the contribution of visible kinematics to judgments of expressivity, emotion, and performance quality? Throughout, the aim is not to claim evidential closure, but to differentiate between well-supported empirical regularities and more speculative theoretical interpretations. To avoid reducing complex cognitive theories to descriptive metaphors, terms such as predictive processing and generative models are used only where studies provide explicit computational or formal mechanistic grounding (Cusimano et al., 2024; Matthews et al., 2026).

### 1.1 Review approach

This article adopts a structured critical narrative review approach rather than a full systematic review or meta-analysis. Relevant literature was identified through targeted searches in Web of Science, Scopus, PsycINFO, and Google Scholar, supplemented by backward and forward citation tracking of key studies. Searches covered publications available up to March 2026 and combined terms related to piano performance, ancillary movement, expressive movement, gesture, audiovisual integration, visual dominance, embodied cognition, and cognitive load. Priority was given to empirical studies directly examining solo piano performance. However, adjacent work from broader music-performance, perception, and cognitive-neuroscience contexts was included when it clarified methodological issues or helped delimit mechanistic claims. For interpretive clarity, the reviewed literature is organized into three broad groups: performer-side kinematic and physiological evidence, audience-side audiovisual-perception studies, and neurophysiological or computational work used primarily to constrain theoretical interpretation rather than to serve as direct evidence for piano-specific mechanisms. The goal is therefore critical synthesis rather than exhaustive coverage.

## 2 Performer-side evidence: kinematic organization, physiological correlates, and the limits of cognitive-scaffolding claims

### 2.1 Kinematic evidence aligned with musical structure: phrasing and meter

Current evidence generally suggests that movements often classified as ancillary can align with aspects of musical structure. For instance, MacRitchie et al. (2009) analyzed motion capture data from pianists, revealing that extreme values in principal body movements often tend to correspond to phrase boundaries, although variability across performers and contexts remains substantial, with movement troughs occasionally lagging slightly behind acoustic phrase endings. Similarly, Thompson and Luck (2012) observed that movement amplitude is not distributed evenly; rather, it concentrates at structurally salient points such as phrase boundaries, accents, and metrical nodes. Expanding on this, Massie-Laberge et al. (2019) identified head and torso displacement as prominent sources of kinematic variance in the performances examined. Consequently, movements often classified as ancillary are not adequately characterized solely as random biomechanical noise; rather, they often exhibit systematic relationships with musical structure and expressive organization. Nevertheless, these movements do not operate as rigid templates. While individual pianists maintain consistent movement contours across multiple repetitions (Davidson, 2007), multi-subject analyses reveal that different performers employ distinct kinematic strategies to achieve similar structural goals (Massie-Laberge et al., 2019; Thompson and Luck, 2012). Thus, the current data supports a more nuanced conclusion: while these movements can serve structurally relevant functions in piano performance, their form and extent remain highly individualized across performers and musical contexts.

### 2.2 Physiological coordination and temporal coupling: breathing, EMG, and postural sway

In contrast to the robust kinematic data, physiological evidence remains sparse and struggles to substantiate strong causal claims regarding cognitive scaffolding. Investigating this, Nakahara et al. (2010) compared pianists under varying expressive and movement-restricted conditions. While expressive versus non-expressive performance differed on several physiological measures, including heart rate, HRV-related indices, valence, and sweating rate, restricting ancillary body movements within expressive performance significantly affected only respiratory rate. This selective sensitivity indicates that such movements are not merely byproducts of physical exertion. Supporting this, Sakaguchi et al. (2016) demonstrated that breathing timing tightly couples with musical events such as tempo fluctuations, rests, and slurs. This question matters because some interpretations implicitly suggest that visible or felt bodily movement may do more than accompany expression—that it may help performers organize timing, phrasing, or attentional resources during performance. However, one recurring—but still weakly substantiated—hypothesis is that such movements may contribute to the regulation of cognitive load, potentially by leveraging bodily dynamics such as posture or rhythmic movement patterns to stabilize temporal or structural representations. Even so, this idea remains largely speculative and is not consistently formalized across the literature. Many current studies predominantly measure movement organization, breathing patterns, or muscle activation. Very few employ dual-task paradigms, pupillary load metrics, or working memory manipulations to assess actual cognitive relief (Mailly et al., 2025; Turner et al., 2021). Therefore, a more empirically grounded stance is that while such movements may facilitate phrase segmentation, timing maintenance, and motor pre-organization, their specific role as a cognitive offloading mechanism remains a speculative hypothesis rather than a validated construct.

### 2.3 Methodological limitations in current performer studies

A primary methodological limitation within this domain is the constrained accumulation and generalizability of data. Foremost, sample sizes are notoriously small, with numerous motion capture studies relying on fewer than 10 participants (Davidson, 2007; Nakahara et al., 2010; Turner et al., 2025). Furthermore, experimental designs frequently utilize brief musical excerpts under highly controlled laboratory conditions. While this approach effectively isolates variables, it severely compromises ecological validity, stripping away the open, interpretive nature of real-world musical performance (Turner et al., 2021). Compounding these issues, studies vary significantly in their chosen motion metrics, data collection methodologies, and units of analysis, thereby complicating cross-study comparisons (Mailly et al., 2025). Crucially, there is a distinct absence of longitudinal evidence; the trajectory of how these movements develop and stabilize during practice and long-term skill acquisition remains unknown. Ultimately, while existing research supports the structural and expressive relevance of movements often discussed under the label of ancillary movements, testing whether they can be interpreted as cognitive scaffolds requires larger sample sizes, enhanced ecological validity, and rigorous longitudinal tracking.

## 3 Audience perspective: audiovisual integration and expressive perception

### 3.1 Visual dominance: the Tsay effect and extensions

Investigations into visual dominance yield some of the most compelling evidence regarding the audience’s perspective. In a comprehensive meta-analysis of 15 studies comprising 1,298 participants, Platz and Kopiez (2012) showed that the inclusion of visual information significantly elevates performance evaluations compared to audio-only conditions, yielding a moderate average effect size (Cohen’s *d* = 0.51, 95% CI [0.42, 0.59]). Thus, visual kinematics serve as salient and often influential cues for esthetic judgments. Tsay (2013) extended this paradigm strikingly, revealing that non-musicians could identify competition winners at above-chance levels (52.5%) using merely 6-s silent videos, whereas audio-only judgments fell to 25.5%. This effect persisted among domain experts, whose video-condition accuracy (46.6–47.0%) vastly outperformed audio-only conditions (20.5–25.7%, *d* = 1.01–1.20). Crucially, accuracy remained robust (48.8%) even when stimuli were reduced to black-and-white point-light motion outlines, suggesting that dynamic kinematic trajectories—rather than static appearance or attire—play an important role in this visual-dominance effect (Platz and Kopiez, 2012; Tsay, 2013; Juchniewicz, 2008).

Nevertheless, visual information does not universally supersede auditory input; recent replication efforts highlight critical boundary conditions. While Mehr et al. (2018) successfully replicated the visual dominance effect using Tsay’s original stimuli, accuracy decreased toward chance levels when novel, comparable videos were introduced. Similarly, Wilbiks and Yi (2022) failed to observe a significant visual advantage. Conversely, by meticulously controlling for repertoire and camera variables, Vogt et al. (2026) reinstated the effect, with video-only accuracy (50.5%) significantly exceeding both audio-only (35.1%) and audiovisual conditions (38.0%, *d*′ = 0.572). Synthesizing these findings suggests that audiences often overweight visual cues, especially under rapid or high-uncertainty evaluative conditions characterized by salient kinematic disparities (Mehr et al., 2018; Vogt et al., 2026; Wilbiks and Yi, 2022).

Furthermore, the moderating role of audience expertise remains inconsistent. While Tsay (2013) and Griffiths and Reay (2018) found that formal musical training did not significantly attenuate the visual bias in quality judgments—with the latter noting that visual cues outweighed auditory ones in conflicting expert-amateur pairings—Samma et al. (2025) reported that visual dominance only manifested when non-wind instrumentalists evaluated wind performances. This suggests that expertise likely moderates audiovisual weighting through task-specific experience matching; observers can resist visual heuristics more effectively when possessing corresponding auditory-motor representations (Griffiths and Reay, 2018; Samma et al., 2025; Tsay, 2013). Moreover, while point-light display paradigms confirm that pure kinematic trajectories transmit substantial expressive information, displays more closely approximating human morphology (e.g., body-mass or skeleton models) facilitate enhanced expressivity evaluations. This indicates that audiences read movement trajectories but utilize full-body contours to optimize attribution (Moura et al., 2023; Tsay, 2013).

### 3.2 Mechanisms of audiovisual integration: processing the cues

Mechanistic research elucidates how audiences integrate these multimodal cues temporally. Employing continuous slider paradigms, Vines et al. (2006) showed that while both audio and video streams convey phrasing information, they modulate perceptual tension divergently. Visual cues appear to shape the experience of tension, prolong phrase perception, and signal impending emotional shifts, potentially functioning as online guiding cues. Corroborating this through physiological measures, Chapados and Levitin (2008) utilized galvanic skin responses to reveal that audiovisual reactions exceed the additive sum of isolated audio and video modalities. Notably, visual cues become particularly salient during acoustic rests, supporting the interpretation that cross-modal integration is taking place (Chapados and Levitin, 2008; Vines et al., 2006).

Beyond online processing, continuous evaluation paradigms reveal that visual cues pre-shape audience judgments even before acoustic onset. Waddell and Williamon (2017) demonstrated that inappropriate stage entrances significantly depress initial evaluations, an effect particularly pronounced and accelerated among musically trained evaluators. While negative facial expressions in isolation do not necessarily decrement final scores, they trigger substantial and enduring rating drops when systematically paired with acoustic errors (*η*^2^ = 0.28, *r* = 0.90). In this context, facial expressions operate as an interpretive framework that amplifies error salience (Waddell and Williamon, 2017). Although eye-tracking research indicates that stage social and structural cues guide audience visual attention—such as fixating on the melody-carrying performer or instances of joint attention in duets (Kawase and Obata, 2016)—direct oculomotor studies isolating solo piano movements commonly discussed as ancillary remain scarce.

### 3.3 Caution in applying predictive-processing frameworks

Predictive processing refers to computational accounts in which the brain continuously generates and updates expectations about incoming sensory input, while generative models specify how such expectations are formally structured and revised (Clark, 2013; Skerritt-Davis and Elhilali, 2021). In the present context, these frameworks are relevant because performer movement may shape what observers expect to hear or see during musical performance.

However, most studies reviewed here do not directly test prediction-error dynamics or implement formal generative models. Rather, they show that visual information can bias judgment, shape tension perception, or increase sensitivity to audiovisual mismatch. These findings are therefore more securely interpreted in terms of audiovisual integration, mismatch sensitivity, or sensorimotor coupling than as evidence for a full predictive-processing account. For this reason, predictive-processing terminology should be used cautiously unless supported by explicit computational modeling or neural evidence (e.g., Cusimano et al., 2024; Skerritt-Davis and Elhilali, 2021).

## 4 Critical synthesis: integrating evidence and identifying methodological gaps

A central interpretive challenge in this literature is that studies differ not only in method, but also in how directly they bear on solo piano performance. A primary issue is that the literature varies widely in levels of evidential directness: while some studies directly examine solo piano movements commonly discussed as ancillary, others are included primarily for their methodological or mechanistic relevance, serving as indirect or constraint-based evidence rather than direct tests of piano-performance mechanisms. Beyond this variance, attempts to validate the cognitive-scaffolding hypothesis and broader embodied-cognition claims expose two fundamental methodological gaps. First, the field demonstrates an overwhelming reliance on correlational observations, leaving the causal mechanisms driving both audience evaluation and performer execution largely untested. Second, a persistent disconnect remains between descriptive embodied metaphors and rigorous neurophysiological data, particularly regarding whether these movements genuinely alleviate specific internal or extraneous cognitive demands. The following subsections dissect these two critical limitations to clarify the necessary trajectory for future experimental designs.

### 4.1 Abundant correlational evidence, scarce causal evidence

More fundamentally, the interpretive difficulty is not only causal but also conceptual: if ancillary movements are treated as an analytical rather than biomechanically discrete category, then causal tests must specify which movement features are being manipulated and why those features are grouped together. The fundamental limitation is not the total absence of causal evidence, but the relative scarcity of tightly controlled causal tests targeting specific ancillary movement features. Motion-capture and physiological studies suggest that movements commonly discussed as ancillary covary with phrase boundaries and breathing, whereas psychophysical research confirms that visual conditions systematically alter audience judgments (Massie-Laberge et al., 2019; Nakahara et al., 2010; Tsay, 2013; Vines et al., 2006). A critical question nevertheless remains insufficiently addressed: Do systematic, artificial alterations of movements commonly discussed as ancillary—while holding sound constant—causally shift audience perception, beyond the broader movement effects already demonstrated in prior work (Juchniewicz, 2008)?

Strict causal evidence specific to ancillary movement features remains scarce. Although Tsay (2013) and Moura et al. (2023) isolated kinematics by removing appearance factors, and others utilized audiovisual asynchrony paradigms to demonstrate mismatch detection (Bishop and Goebl, 2014; Proverbio and Valtolina, 2025), these studies do not isolate the specific causal contribution of movement features commonly discussed as ancillary, nor do they clearly establish which kinematic components drive changes in perceived expressivity or structural understanding. The field would benefit from more artificial manipulation paradigms capable of establishing clearer manipulation-response chains. Future research could employ motion-captured piano virtual avatars, systematically manipulating head nods or torso sway against a fixed audio track to assess audience ratings. Alternatively, generative video editing (e.g., deepfakes) offers precise kinematic manipulation without acoustic alteration. While synchronous nodding affects ensemble coordination in existing VR studies (Van Kerrebroeck et al., 2021), such high-control manipulation paradigms remain relatively scarce, although recent work (Ashkenazi, 2021) has begun to address this gap by experimentally isolating the causal influence of visual information on auditory perception. Consequently, while movements commonly discussed as ancillary are often associated with expressive performance, the underlying causal mechanisms remain insufficiently specified.

### 4.2 The gap between embodied metaphors and neurophysiological data

Furthermore, the field frequently overextends the term “embodied cognition.” Observing movement-structure synchronization and immediately invoking embodiment—without direct neural or computational evidence—reduces a complex framework to a catch-all metaphor for missing mechanistic explanations. Current literature suggests that bodily factors matter, yet it does not establish that these movements are integral to cognitive processing rather than reflecting technical inertia or postural compensation.

This gap is particularly evident in neurophysiological research. While mobile EEG suffers from severe motion artifacts during real performance, functional near-infrared spectroscopy (fNIRS) better tolerates movement in naturalistic music contexts (Curzel et al., 2024; Tervaniemi, 2023). However, few studies integrate motion capture, movement restriction, and cortical measurement; existing fNIRS and EEG piano studies primarily explore sight-reading versus improvisation or monitor baseline task load (Beauchemin et al., 2024; Kang et al., 2025). Interpreting such movements as cognitive scaffolds on the basis of performer movement alone remains insufficiently supported without multimodal validation. Future studies must translate embodiment into testable hypotheses by comparing free and movement-restricted playing within the same pianist, simultaneously recording kinematics, neuroimaging, and pupillary metrics. If restriction increases prefrontal load or compromises metrical stability while audio remains matched, such movements would be more plausibly interpreted as participating in cognitive processing. Conversely, if restriction solely alters external posture without inflating cortical load, the mechanism is likely biomechanical.

## 5 Conclusion and future directions

Current evidence supports one comparatively stronger conclusion and one more tentative one. The stronger conclusion concerns audience perception: visible performer movements often shape judgments of expressivity, emotion, and performance quality. A more cautious conclusion concerns performers: existing studies support movement–structure coupling more clearly than cognitive offloading (Massie-Laberge et al., 2019; Moura et al., 2023; Tsay, 2013).

Nevertheless, the field should move beyond treating correlational observations as if they already established specific mechanisms. Important open questions remain regarding how movements often classified as ancillary develop during skill acquisition, whether they are associated with cognitive-load reduction, and how audience responses may relate to predictive accounts at the neural level. To address these gaps, future research should prioritize two trajectories. First, implement semester-long longitudinal motion capture studies on piano students, utilizing mixed-effects models to track how proficiency and time impact movement stability. Second, execute systematic kinematic manipulations in VR or generative video environments with fixed audio streams, employing continuous scoring and eye-tracking to isolate the visual parameters driving audience judgments. Integrating these manipulation designs with mobile EEG or fNIRS may help advance ecological music cognition research from descriptive interpretation toward more directly testable mechanistic accounts (Curzel et al., 2024; Tervaniemi, 2023; Van Kerrebroeck et al., 2021). More broadly, rather than treating ancillary movements as a clearly bounded category, future research may benefit from conceptualizing them as emergent properties of whole-body coordination systems, shaped jointly by biomechanical constraints, expressive intentions, and perceptual affordances. From this perspective, the term ancillary movements may remain heuristically useful, provided it is treated as a functional and interpretive shorthand rather than a sharply bounded biomechanical category.
